# Acute Aortic Dissection Biomarkers Identified Using Isobaric Tags for Relative and Absolute Quantitation

**DOI:** 10.1155/2016/6421451

**Published:** 2016-06-15

**Authors:** Ziya Xiao, Yuan Xue, Chenling Yao, Guorong Gu, Yaping Zhang, Jin Zhang, Fan Fan, Xiao Luan, Zhi Deng, Zhengang Tao, Zhen-ju Song, Chaoyang Tong, Haojun Wang

**Affiliations:** Department of Emergency, Zhongshan Hospital, Fudan University, Shanghai 200032, China

## Abstract

The purpose of this study was to evaluate the utility of potential serum biomarkers for acute aortic dissection (AAD) that were identified by isobaric Tags for Relative and Absolute Quantitation (iTRAQ) approaches. Serum samples from 20 AAD patients and 20 healthy volunteers were analyzed using iTRAQ technology. Protein validation was performed using samples from 120 patients with chest pain. A total of 355 proteins were identified with the iTRAQ approach; 164 proteins reached the strict quantitative standard, and 125 proteins were increased or decreased more than 1.2-fold (64 and 61 proteins were up- and downregulated, resp.). Lumican, C-reactive protein (CRP), thrombospondin-1 (TSP-1), and D-dimer were selected as candidate biomarkers for the validation tests. Receiver operating characteristic (ROC) curves show that Lumican and D-dimer have diagnostic value (area under the curves [AUCs] 0.895 and 0.891, *P* < 0.05). For Lumican, the diagnostic sensitivity and specificity were 73.33% and 98.33%, while the corresponding values for D-dimer were 93.33% and 68.33%. For Lumican and D-dimer AAD combined diagnosis, the sensitivity and specificity were 88.33% and 95%, respectively. In conclusion, Lumican has good specificity and D-dimer has good sensitivity for the diagnosis of AAD, while the combined detection of D-dimer and Lumican has better diagnostic value.

## 1. Introduction

Acute aortic dissection (AAD) is a common and devastating disease with high disability and death rates. Various etiologies can cause aortic intimal injury, and bleeding through the intimal media into aortic media leads to vascular wall stratification. The per-hour mortality rate in untreated patients is as high as 1% [1]. Unfortunately, there is no widely available, cost-effective biomarker assay for AAD with high sensitivity and specificity, underscoring the need for rapid and economical diagnostic methods. The isobaric Tags for Relative and Absolute Quantitation (iTRAQ) is an effective quantitative proteomic assay for low-abundance proteins that has been utilized to identify biomarkers for various disease conditions [2]. Using this method, we set out to determine serum biomarkers released following disruption of the aortic media that can provide sufficient specificity and sensitivity for diagnosing AAD.

## 2. Materials and Methods

### 2.1. Clinical Data

A total of 60 AAD patients (AAD group) and 60 non-AAD patients (non-AAD group) who presented to Zhongshan Hospital Fudan University (Shanghai, China) within 72 hours after sudden onset of chest and/or back pain lasting 5 minutes or more were enrolled. Inclusion criteria were as follows: (1) age: greater than or equal to 18 years of age; (2) no gender restriction; (3) patients who have chest pain and at the onset within 72 h; and (4) patients with acute aortic dissection that should be confirmed by the aortic computed tomographic arteriography (CTA) or angiography. Exclusion criteria were as follows: (1) women during pregnancy or lactation; (2) the history of cardiopulmonary resuscitation (CPR) and history of cardiac interventional therapy or surgery in 1 week; (3) severe liver and renal insufficiency patients; and (4) patients in a state of shock who need to use vasoactive drugs at admission to the hospital. The time frame for inclusion of patients was from 0.5 h to 72 h. The mean age of AAD patients was 56.88 ± 11.65 years. The non-AAD patients suffered from ST-segment elevation myocardial infarction (STEMI, *n* = 11), non ST-segment elevation myocardial infarction (NSTEMI, *n* = 16), unstable angina pectoris (*n* = 14), pulmonary embolism (*n* = 5), pneumonia (*n* = 2), duodenal ulcer (*n* = 1), esophagitis (*n* = 1), and non-AAD chest pain of unknown origin (*n* = 10). Their mean age was 56.85 ± 13.23 years. An additional 60 patients without a history of cardiovascular disease (CVD) were randomly selected from subjects who underwent outpatient health examinations during the same period to comprise a control group. The mean age was 52.68 ± 6.77 years. The first 20 cases were selected from both the AAD and control groups for iTRAQ analysis. Whole blood samples were immediately collected after admission, allowed to stand at room temperature for 1 hour, and centrifuged at 4000 rpm for 10 min. The serums were then aliquoted and stored at −80°C until analysis. The study was performed according to the principles of the Declaration of Helsinki and was approved by the Ethics Committee of Zhongshan Hospital.

### 2.2. Experimental Methods

#### 2.2.1. iTRAQ Sample Preparation: Strong Cation Exchange (SCX) Chromatography

iTRAQ reagents were purchased from Applied Biosystems (Foster City, CA, USA). Fourteen interfering highly abundant proteins from serum samples were removed using Agilent multiple affinity removal liquid chromatography (LC) column-Human 14 (MARS) (Shimadzu, Kyoto, Japan). Next, 100 *μ*g of each extract was precipitated using acetone at −20°C and suspended in 20 *μ*L Dissolution Buffer (Applied Biosystems). After reduction and alkylation, each sample was digested with trypsin (w[trypsin] : w[protein] = 1 : 20) at 37°C overnight. The tryptic peptides were labeled with the iTRAQ reagents; the AAD and control groups were labeled with iTRAQ 113 and 114, respectively. The peptides were pooled and desalted with Sep-Pak Vac C18 (Waters, Milford, MA, USA). The peptide mixture was diluted with Buffer A containing 10 mM KH_2_PO_4_ in 25% acetonitrile (ACN) at pH 2.6. The peptides were fractionated by a 20AD high-performance liquid chromatography (HPLC) system (Shimadzu) equipped with polysulfoethyl A column (2.1 mm × 100 mm, 5 *μ*m, 200 A, The Nest Group, Southborough, MA, USA). The composition of Buffer B was 350 mM KCl, 10 mM KH_2_PO_4_, and 25% ACN at pH 2.6. Separation was performed using a linear binary gradient of 0–80% Buffer B in Buffer A at a flow rate of 200 *μ*L/min for 1 hour. The fractions were combined into 20 groups.

#### 2.2.2. LC-MS Analysis

Each SCX fraction was dried down with a rotary vacuum concentrator, dissolved in Buffer C (0.1% formic acid, 5% ACN, and 95% water) and analyzed on Qstar XL (Applied Biosystems). The HPLC gradient was 5–35% Buffer D (95% ACN, 0.1% formic acid) in Buffer C at a flow rate of 300 nL/min for 70 min. Analysis survey scans were acquired with mass spectrometry (MS) from* m/z* 400–1800 with up to four precursors selected for MS/MS from* m/z* 100–2000.

#### 2.2.3. Biomarker Verification

D-dimer was detected by a Sysmex CS-2000i automatic coagulation instrument. C-reactive protein (CRP) was detected by a Vitros 5.1 FS automatic biochemical analyzer (Johnson & Johnson, New Brunswick, NJ, USA). Lumican and thrombospondin-1 (TSP-1) were detected by enzyme-linked immunosorbent assay (ELISA), using a Lumican kit (Biomatik Company, Cambridge, ON, Canada) and a TSP-1 kit (R&D Systems Company, Minneapolis, MN, USA). The operations were performed in strict accordance with the manufacturers' instructions. The kit-provided original standards were used in gradual dilution, and the standard curves were used to calculate biomarker concentrations in the samples. Each sample was tested in duplicate.

### 2.3. Statistical Analysis

MS data were analyzed on ProteinPilot 3.0 (AB SCIEX, Foster City, CA, USA). Only peptides identified with confidence interval ≥95% (Unused ProtScore > 1.3) were used for protein identification compilation and quantitation calculation. Fold changes >1.2 or <0.8 were set as cut-off values to designate significant differences in protein expressions between the AAD and control groups. The functional information of proteins was obtained by retrieval from Uniprot. All statistical analyses were performed on SPSS 17.0 (SPSS Inc., Chicago, IL, USA) and GraphPad Prism 5 (GraphPad Inc., La Jolla, CA). Results are presented as mean ± standard deviation (SD). Multiple groups were compared with independent sample *t*-test, one-way analysis of variance (ANOVA), or chi-square test. Statistical significance was defined as *P* < 0.05. The roles of individuals and joint detection of candidate biomarkers were analyzed by receiver operator characteristic (ROC) curves and logistic regression modeling.

## 3. Results

### 3.1. Clinical Features of iTRAQ and Validation Analysis

The first 20 samples from AAD and control groups were used for iTRAQ analysis. The clinical features of the two groups are summarized in Table 1. There was no significant difference in age distribution or sex composition between two groups (*P* > 0.05).

Validation testing was performed for 60 AAD patients, 60 non-AAD patients, and 60 healthy volunteers. The clinical features of the three groups are summarized in Table 2. There was no significant difference in age distribution or sex composition among the three groups (*P* > 0.05). There was no significant difference in the time from symptom onset to admission or the numbers of hypertension cases between the AAD and non-AAD groups (*P* > 0.05).

### 3.2. Identification Results and Functional Classification of Serum Proteome

**Figure 1:**
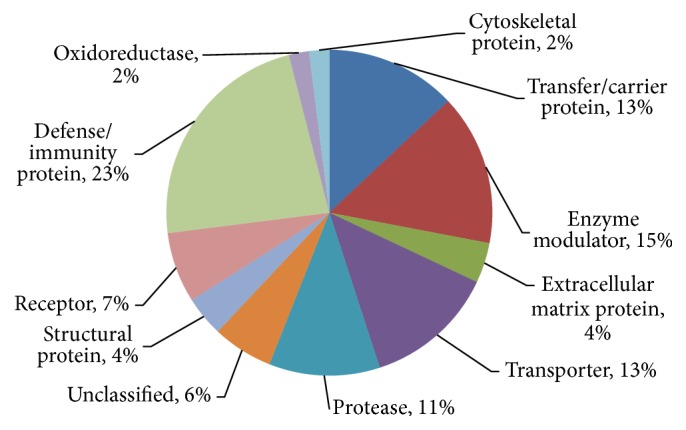
Identification results and functional classification of the serum proteome.

A total of 355 proteins were identified by MS, and 164 met the strict quantitative standard. Uniprot comment information was used to analyze the differentially expressed serum proteins of iTRAQ, and 164 proteins were classified based on their biofunctions. The major types include defense/immunity protein (23%), enzyme modulator (15%), transfer/carrier protein (13%), transporter (13%), protease (11%), receptor (7%), ECM protein (4%), structural protein (4%), oxidoreductase (2%), cytoskeletal protein (2%), and unclassified types (6%) (Figure 1).

Among the 164 proteins with a relative quantitation difference for AAD patients compared with normal controls, 64 and 62 proteins increased and decreased more than 1.2-fold among the AAD patients, respectively. Among the identified proteins with differential expression, there were a number of acute phase reactants, blood coagulation proteins, and extracellular matrix (ECM) proteins (Table 3).

### 3.3. Validation of Candidate Molecular Markers

Based on the iTRAQ findings and previous interesting protein reports [3–6], we selected Lumican (ECM protein), CRP (an acute phase reactive protein), TSP-1 (a blood coagulation protein), and D-dimer as target biomarkers for verification. The differential expressions of the four proteins among the AAD (*n* = 60), non-AAD (*n* = 60), and control (*n* = 60) groups are shown in Table 4.

### 3.4. Diagnostic Values of Lumican, CRP, TSP-1, and D-Dimer for AAD

**Figure 2:**
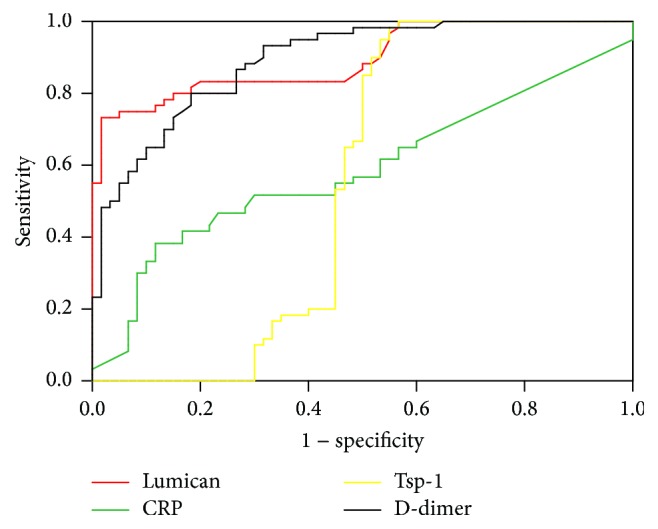
ROC curves for diagnosing AAD by Lumican, CRP, TSP-1, and D-dimer.

Using data from the AAD and non-AAD groups, ROC curves were plotted for the use of Lumican, CRP, TSP-1, and D-dimer in diagnosing AAD, selecting Youden index maximum value as the cut-off value to get the appropriate sensitivity and specificity (Figure 2 and Table 5).

### 3.5. Diagnostic Value of Lumican and D-Dimer Combined Detection in Diagnosis of AAD

#### 3.5.1. Logistic Regression Analysis of Biological Markers for Detection and Diagnosis of AAD

The ROC curves of the four biomarkers show that Lumican and D-dimer have significant values for AAD diagnosis (*P* < 0.05). A combined forecasting model was established using the detection results of Lumican and D-dimer as independent variables and disease state as the dependent variable: logit(*P*) = −5.127 + 2.151 × Lumican + 0.296 × D-dimer. Then, the variables and statistics were substituted into the model (Table 6).

#### 3.5.2. ROC Curve Analysis of Lumican and D-Dimer

**Figure 3:**
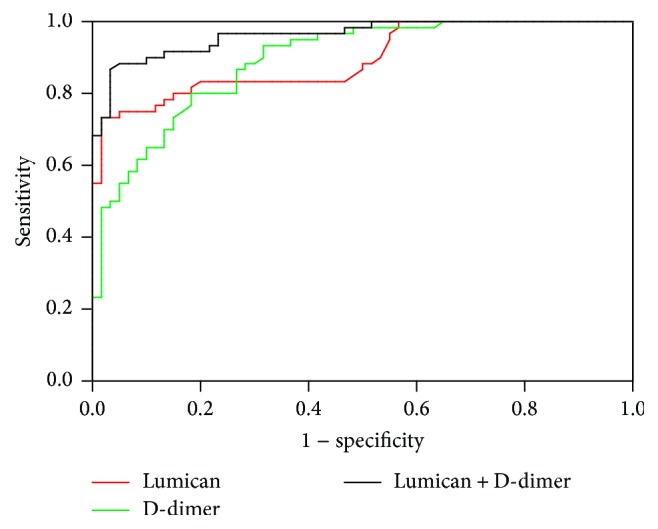
ROC curves for AAD diagnosis using Lumican and/or D-dimer detection.

The detection results of Lumican and D-dimer were substituted into the model: −5.127 + 2.151 × Lumican + 0.296 × D-dimer. The ROC curves were analyzed using the modeling results as a new variable *Y*. Lumican and D-dimer combined detection had higher diagnostic value (AUC = 0.962, *P* < 0.01) than either protein alone, with 88.33% sensitivity and 95% specificity (Figure 3).

## 4. Discussion

AAD is a life-threatening cardiovascular emergency with a mortality rate of 1-2% per hour soon after symptom onset. The missed diagnosis rate of AAD in emergency rooms is up to 38%, and 28% of AADs are diagnosed by autopsy [7]. The cause of death in AAD patients is related to disease severity and development, as well as poor blood pressure control. Delayed diagnosis dramatically increases the mortality risk. There are numerous clinical symptoms and signs of AAD, but its diagnosis relies on large imaging equipment, which increases the costs and requires well-trained technical personnel. Thus, AAD cannot be diagnosed in many small and medium hospitals. Many patients die or become disabled because of aortic rupture or other serious complications before diagnosis. Therefore, it is urgent to identify rapid, noninvasive, economical, and effective biomarkers to diagnose or exclude AAD.

First reported in 2004, iTRAQ is a relatively new isotope labeling technology that is widely used to screen many disease biomarkers [8, 9]. Here, iTRAQ was used to compare the serum proteomes between the AAD and control groups. A total of 125 differentially expressed proteins were identified to have at least 1.2-fold changes, including ECM proteins, acute phase reactive proteins, and blood coagulation proteins. Lumican is ECM protein that can be produced by aortic smooth muscle cells [10] and it may potentially be associated with vascular injury, and CRP and TSP-1 showed the highest fold change in acute phase reactive proteins and blood coagulation proteins, respectively. For verification, we selected Lumican, CRP, and TSP-1 as promising candidates from the three categories listed above, as well as D-dimer, which has known diagnostic value. The results showed significantly higher serum expression levels of D-dimer, CRP, TSP-1, and Lumican in the AAD group versus the control group (*P* < 0.05), which is consistent with the MS data. The expressions of Lumican, CRP, and D-dimer were also significantly higher compared with the non-AAD group, but TSP-1 expression was decreased, indicating that it is not likely useful for diagnosing AAD.

D-dimer is a specific fibrinolytic marker produced by degradation of fibrin monomer by factor XIII. Increased plasma D-dimer levels are suggestive of thrombosis and fibrinolysis that can occur following disseminated intravascular coagulation (DIC), pulmonary embolism, cerebral infarction, acute myocardial infarction, and other diseases. AAD will lead to injury and subsequent aortic release tissue factor, formation of false lumen thrombosis, endogenous coagulation reaction, and activation of the fibrin dissolution system, causing the release of D-dimer into peripheral blood. In fact, D-dimer is already used as a biomarker in AAD diagnosis [11, 12]. We confirmed that serum D-dimer contents in the AAD group were significantly higher than in the non-AAD and control groups (13.48 ± 20.75 versus 1.62 ± 2.62 versus 0.12 ± 0.06 mg/L, *P* < 0.05), with a cut-off value of 1.435 mg/L, 93.33% sensitivity, and 68.33% specificity, showing high diagnostic sensitivity for AAD.

Lumican is a small leucine-rich proteoglycan and an important ECM component of the aortic wall. It can be produced by aortic smooth muscle cells [10] and is related to collagen fiber arrangement and growth. Lumican plays important roles in cell proliferation and migration and differentiation and tissue repair [13]. It is also associated with cardiovascular remodeling [14]. ECM proteins may be closely related to AAD pathogenesis and is therefore a potential biomarker [15]. Our previous study showed that Lumican may be useful for diagnosing AAD [3]. The present investigation confirms that serum Lumican levels are significantly higher in the AAD group compared to the non-AAD and control groups (3.39 ± 1.66 versus 1.12 ± 0.56 versus 0.42 ± 0.31 ng/mL, *P* < 0.05), with AAD cut-off value of 2.19 ng/mL, 73.33% sensitivity, and 98.33% specificity.

CRP is an acute phase protein synthesized by liver cells under inflammatory stimuli such as microbial invasion or tissue injury. As a marker of inflammation, CRP is closely related to many CVDs such as hypertension, atherosclerosis, coronary heart disease, and myocardial infarction. Increased CRP levels correlate with the development of false lumen thrombosis in AAD patients [16], which is an independent risk factor for predicting mortality [17, 18]. TSP-1 is a multifunctional glycoprotein widely distributed in blood, heart, cartilage, lung, and brain and it is involved in important physiological processes. This endogenous molecule can inhibit new blood vessel formation [19] and promote platelet activation and aggregation [20]. However, the ROC curves revealed that neither CRP nor TSP-1 had diagnostic value for AAD (*P* > 0.05).

Although single serum markers can help in diagnosis of AAD, their sensitivities and specificities are far below ideal levels. Nevertheless, combined detection of multiple indicators is one way to improve the clinical value of diagnostic tests. Because D-dimer is a high sensitivity and low-specificity protein and Lumican is a high-specificity and low-sensitivity protein, their combined use has higher sensitivity, specificity, and accuracy for diagnosing AAD. Therefore, we investigated the diagnostic values from the independent and combined use of these two biomarkers for AAD detection. We plotted ROC curves to reflect the sensitivity and specificity, and logistic regression modeling was carried out to fit the two biomarkers together. The area under the new ROC curve was 0.962, with 88.33% sensitivity and 95% specificity. These results indicate that combined Lumican and D-dimer detection optimizes sensitivity and specificity to improve AAD diagnosis accuracy.

In summary, the combined detection of Lumican and D-dimer could help clinicians diagnose AAD in emergency settings lacking advanced imaging equipment. Lumican is ECM protein that reflects aortic wall injury and repair, while D-dimer increases are indicative of excessive fibrinolysis following AAD. Lumican and D-dimer are therefore complementary biomarkers for AAD diagnosis. However, our sample size is relatively small, and the diagnostic value and prognostic significance of these biomarkers for AAD should be verified in future studies.

## 5. Conclusions

iTRAQ is a suitable approach for identifying AAD biomarkers. The Lumican and D-dimer assays are highly specific and sensitive methods, respectively, for the diagnosis of AAD. Combining the two assays can therefore help clinicians diagnose or rule out AAD.

## Figures and Tables

**Table 1:** Clinical features of the iTRAQ analysis subjects.

	AAD group	Normal controls	*P* value
*n*	20	20	/
Age (mean ± SD)	60.95 ± 12.74	55.5 ± 8.62	0.1214^a^
Gender (male/female)	14/6	14/6	1^b^
Admission after onset hours (mean ± SD)	5.18 ± 3.16	/	/
Stanford type A/B (*n*)	12/8	/	/
Marfan syndrome (*n*)	0	0	/
Hypertension (*n*)	11	/	/

^a^ *t*-test. ^b^Chi-square test.

**Table 2:** Clinical features of the validation analysis subjects.

	AAD group	Non-AAD group	Normal controls	*P* value
*n*	60	60	60	/
Age (mean ± SD)	56.88 ± 11.65	56.85 ± 13.23	52.68 ± 6.77	0.0551^a^
Gender (male/female)	43/17	49/11	39/21	0.1187^b^
Admission after onset hours (mean ± SD)	22.51 ± 19.71	16.73 ± 17.57	/	0.0926^c^
Stanford type A/B (*n*)	39/21	/	/	/
Marfan syndrome (*n*)	2	0	0	0.1323^b^
Hypertension (*n*)	34	31	/	0.7142^b^

^a^One-way ANOVA, ^b^chi-square test, and ^c^ *t*-test.

**Table 3:** Subset of differentially expressed proteins between the AAD and control groups.

*N*	Accession	Name	Biological process	Protein class	AAD : CON	Upregulated/downregulated
1	P02748	Complement component C9	Response to stimulus	Receptor	1.9231	Up
2	P51884	Lumican	Cell-cell adhesion	Receptor	1.4191	Up
3	P00450	Ceruloplasmin	Blood coagulation	Transporter	1.9055	Up
4	P00751	Complement factor B	Blood coagulation	Transfer/carrier protein	1.3932	Up
5	P02741	CRP	Response to stress	Defense/immunity protein	7.379	Up
6	P00738	Haptoglobin	Blood coagulation	Protease	0.2535	Down
7	P02649	Apolipoprotein E	Lipid metabolic process	Transporter	0.3565	Down
8	P04114	Apolipoprotein B-100	Lipid metabolic process	Transfer/carrier protein	0.3767	Down
9	P07996	TSP-1	Blood coagulation	Transfer/carrier protein	0.4699	Down
10	P01019	Angiotensinogen	Protein metabolic process	Enzyme modulator	0.5395	Down

This table lists the five highest Unused ProtScores from the upregulated proteins and downregulated proteins. AAD, acute aortic dissection. CON, normal controls.

**Table 4:** Comparison of Lumican, CRP, TSP-1, and D-dimer serum concentrations (mean ± SD).

	AAD group	Non-AAD group	Normal controls
Lumican (ng/mL)	3.39 ± 1.66	1.12 ± 0.56^*∗*^	0.42 ± 0.31^*∗*^
CRP (mg/L)	35.17 ± 38.61	19.01 ± 25.17^*∗*^	5.13 ± 2.06^*∗*^
TSP-1 (ng/mL)	6052.99 ± 1657.3	6995.38 ± 8053.64	798.49 ± 930.6^*∗*^
D-dimer (mg/L)	13.48 ± 20.75	1.62 ± 2.62^*∗*^	0.12 ± 0.06^*∗*^

Compared with the non-AAD group, serum levels of Lumican, CRP, and D-dimer were significantly higher in the AAD group (^*∗*^ *P* < 0.05), while serum levels of TSP-1 were not significantly different (*P* > 0.05). Compared with the control group, the serum levels of Lumican, CRP, TSP-1, and D-dimer were significantly higher in the AAD group (^*∗*^ *P* < 0.05).

**Table 5:** The diagnostic efficiency analysis of four AAD biomarkers.

	AUC	*P* value	95% CI	Sensitivity (%)	Specificity (%)	Cut-off value	Youden index
Lumican	0.895	<0.01	0.839–0.951	73.33	98.33	2.19 ng/mL	0.7167
CRP	0.586	0.1037	0.482–0.69	38.33	88.33	36.8 mg/L	0.2666
TSP-1	0.551	0.3342	0.434–0.669	98.33	45	2564.5 ng/mL	0.4333
D-dimer	0.891	<0.01	0.836–0.947	93.33	68.33	1.435 mg/L	0.6167

**Table 6:** Logistic regression analysis results of AAD diagnosis with combined Lumican and D-dimer detection.

	*B*	SE	Wald	*P* value	OR	95% CI
Lumican	2.151	0.504	18.188	<0.01	8.592	3.197–23.086
D-dimer	0.296	0.098	9.051	<0.01	1.345	1.109–1.631
Constant	−5.127	0.961	28.433	<0.01	0.006	
